# Modeling Time-Dependent Behavior of Concrete Affected by Alkali Silica Reaction in Variable Environmental Conditions

**DOI:** 10.3390/ma10050471

**Published:** 2017-04-28

**Authors:** Mohammed Alnaggar, Giovanni Di Luzio, Gianluca Cusatis

**Affiliations:** 1 Rensselaer Polytechnic Institute, Troy, NY 12180, USA; alnagm2@rpi.edu; 2 Politecnico di Milano, Milan 20121, Italy; giovanni.diluzio@polimi.it; 3 Northwestern University, 2145 Sheridan Road, Tech A125, Evanston, IL 60208, USA

**Keywords:** Alkali Silica Reaction, Lattice Discrete Particle Model, concrete, Creep, shrinkage, aging, deterioration

## Abstract

Alkali Silica Reaction (ASR) is known to be a serious problem for concrete worldwide, especially in high humidity and high temperature regions. ASR is a slow process that develops over years to decades and it is influenced by changes in environmental and loading conditions of the structure. The problem becomes even more complicated if one recognizes that other phenomena like creep and shrinkage are coupled with ASR. This results in synergistic mechanisms that can not be easily understood without a comprehensive computational model. In this paper, coupling between creep, shrinkage and ASR is modeled within the Lattice Discrete Particle Model (LDPM) framework. In order to achieve this, a multi-physics formulation is used to compute the evolution of temperature, humidity, cement hydration, and ASR in both space and time, which is then used within physics-based formulations of cracking, creep and shrinkage. The overall model is calibrated and validated on the basis of experimental data available in the literature. Results show that even during free expansions (zero macroscopic stress), a significant degree of coupling exists because ASR induced expansions are relaxed by meso-scale creep driven by self-equilibriated stresses at the meso-scale. This explains and highlights the importance of considering ASR and other time dependent aging and deterioration phenomena at an appropriate length scale in coupled modeling approaches.

## 1. Introduction

Alkali-Silica Reaction (ASR), a problem of world wide nature [1], leads to internal deterioration in the form of a distributed network of cracks. In the early stages, the cracks are very fine and usually unseen by naked eye since only cracks with opening >100 μm can be visually noticeable, yet deterioration is enough to reduce concrete strength by a noticeable level [2]. It must be observed here that such deterioration is often overlooked in typical experiments simply because it is counterbalanced by strength increase due to cement hydration While many research efforts are directed towards a permanent cure for ASR affected structures, the currently available solutions are only in the research development stage and have many limitations. Therefore, unfortunately, unless moisture in concrete is reduced below 60% to 80% and maintained below these limits, affected structures, even if rehabilitated, will not stop deteriorating and, eventually, they will need to be replaced.

Basic mechanisms of ASR can be summarized as follows: alkali available in Portland cement react with the silica in siliceous aggregate and produce the so-called basic ASR gel. While the basic ASR gel is believed not to cause expansion [3], it imbibes water and, consequently, it expands resulting in internal pressure inside the concrete meso-structure ultimately causing cracking and damage.

Many variables related to concrete chemistry and composition as well as structural details affect ASR evolution, but a major player is the effect of the surrounding environment. Many researchers confirmed that for ASR to occur, a minimum moisture content must be present, and they reported different critical values for the internal relative humidity, between 60% and 85% [4,5,6,7,8]. In addition, similarly to many chemical reactions and diffusion processes, temperature accelerates the reaction in warmer places [1] and slows it down in colder ones. Furthermore, over the service lifetime—expected to be 100 years according to the most recent guidelines– of concrete structures, other aging and deterioration phenomena interfere and interact with ASR. These include creep, shrinkage [9,10,11], delayed ettringite formation [12,13], freeze-thaw [14], and others. Such complexity limits the validity of typical laboratory approaches for which, one of the main challenges in extrapolating accelerated lab experimental data to real structures is the inconsistency of the acceleration rate of different phenomena, e.g., ASR, creep and concrete aging. For example, creep deformations occurring over multi-decades are believed to relieve ASR expansive pressure in a different way compared to lab accelerated experiments that start and finish at a relatively young age of concrete [15]. The only reasonable way to extrapolate from lab to real applications is through the use of computational models.

The simulation of ASR induced expansion and its effect on both concrete materials and structures has been approached by various authors at different time and length scales with a wide range of levels of sophistication and complexity.

Existing models often even differ on the fundamental assumptions related to ASR mechanisms. Such large variety can be traced back to: (1) specific application needs, e.g., models intended for use in design or retrofit are different from those used to understand the chemistry or replicate the reaction kinetics; (2) experiments used in model development, which have a major effect on the formulated hypothesis and the resulting simplifying assumptions; (3) available computational power which, if limited, restricts the modeling approach to macroscopic level finite element simulations, or whereas, if substantial, allows formulating models all the way to molecular dynamics. Item 2 is likely to be the most important because ASR processes depend highly on the chemistry and mineralogy of aggregate including its silica chemistry, distribution, and content, and it also highly depends on the surrounding binder chemistry including cement, cement replacement products (slag, silica-fume, fly ash, etc.), and additives (superplasticizers). The composition of all these products has huge geographical variations making observations and conclusions obtained in specific research difficult to extrapolate [16,17,18]. This, in turn, limits the general applicability of deterioration models to different experimental observations. On the aggregate side, inside each aggregate particle, the silica distribution is, in most cases, non-uniform and it forms pockets, veins, and scattered inclusions [19,20,21]. Outside the aggregate particle, a variety of different alkali (Na${}^{+}$,K${}^{+}$,Ca${}^{+}$,...) ions are available and all react with silica inside the aggregate in presence of hydroxide (OH${}^{-}$) ions and water (H${}_{2}$O) [17,22,23] that are mainly provided through the cement paste [24,25] especially at later concrete ages [26,27]. With such a variety in both aggregate mineralogy and alkali available ions, the reaction of silica gives rise to an amorphous gel whose precise chemical composition varies widely [28,29]. In all situations, regardless of the chemistry and physics of the reaction and products, the observed result is significant cracking in reactive mixes both in the cement paste and inside the aggregate particles [18,30,31]. Furthermore, experimental (especially petrographic) observations clearly indicate the presence of ASR gel at the aggregate surface (“reaction rim”), inside aggregate particles, and, only in the case of very reactive aggregate, inside cracks.

Several theoretical models were formulated to describe ASR gel evolution as a function of petrographic measurements of aggregate and mortar [3,32,33,34,35,36,37,38,39]. They mainly captured various aspects of ASR expansion including aggregate pessimum size, ASR induced expansion and pressure, but they missed the underlying fracture mechanics of the deterioration process. Bažant [40] predicted the pessimum size of aggregate in a fracture mechanics based formulation and Gao et al. studied the combined pessimum size and specimen size [41]. Dunant and Scrivener recently showed that the effects can be explained by the difference in rate of aggregate reaction that produces early cracking in small aggregate and late cracking in cement paste [42].

At the macroscopic scale, models aimed at describing the global expansion and mechanical deterioration due to ASR. Purely phenomenological models were presented by Charlwood et al. [43] and Thompson et al. [44]. Leger et al. presented a more refined FE model for dam analysis [45] and, later, others included creep [46]. Such models were able to predict well displacements and stress history in the structure, but they completely lack the ability to predict crack patterns and had no connection between the deterioration of mechanical properties and ASR physical phenomena. To improve these models, chemo-mechanical coupling was introduced by Huang and Pietruszczak [47,48] and Ulm et al. [49] who developed models based on the ASR kinetics. Using these models [49] within a smeared crack finite element framework, Farage et al. [50] and Fairbairn et al. [51] were able to reproduce some ASR expansion data available in the literature.

More advanced models considered stress state effects. The model by Saouma and Perotti [52] introduced a three dimensional weighing function describing the dependence of expansion on the stress state. Multon et al. [53] accounted for the shrinkage and compressive strains in beams affected by ASR. Comi et al. [54,55] proposed damage models that combined, in a consistent thermodynamic fashion, the chemical and mechanical components of the ASR process. Furthermore, an important improvement was added by Poyet et al. [56] as he incorporated the effect of humidity and temperature in the reaction kinetics law. A similar, yet more recent, work by Pesavento et al. [57] introduced the humidity effects as a change of the ASR kinetics model by Ulm et al. [49] which originally considered only temperature effects. While all previous models were deterministic, Capra and Sellier [58] formulated a probabilistic model based on the main parameters affecting ASR. For very extensive literature reviews of available ASR models, the reader may want to consult Refs. [1,15,59,60].

All aforementioned models while were successful to some extent, they all lacked the ability to reproduce physically realistic cracking both in pattern and in distribution. They all depended on some sort of phenomenological assumptions and relationships to replicate the degradation effect of ASR. Many models were only simulating expansion, and damage was just a byproduct of restraining and, as a result, such models can never simulate degradation of free expansion tests. In addition, those models which were successful in reproducing stress state effects on ASR expansion, failed to explain complex triaxial behavior of concrete under ASR and had to merely use phenomenological relationships between ASR gel expansion and stress state. A common reason for such limitations is considering concrete as an isotropic, homogenous continuum.

In the literature, it is very rare to find reliable concrete models that incorporate its heterogeneous and random nature by describing it as a multi-phase material (three phase consisting of aggregate, binder and interfacial transition zone or two phase consisting of aggregate and binder). One of the attempts in this direction is the model by Comby-Peyrot et al. [61] which modeled concrete behavior at mesoscale with the application to ASR in a 3D computational framework, which replicated well concrete fracture up to the peak but was unable to reproduce complete degradation in the softening regime. A microscale 2D model by Dunant et al. [62] qualitatively reproduced material deterioration of concrete properties by simulating expansive gel pockets inside the aggregates. Inability to reproduce diffusion of alkali into the aggregate and the simplified 2D character of the model, did not allow the model to produce quantitative results. With the help of scanning electron microscopy techniques, Shin and colleagues [63,64] developed a refined, and computationally very intensive, 2D finite element models of damaged internal structure of concrete. Still in 2D, the micromechanical model by Giorla et al. also included creep effects [11]. This model also suffered from 2D limitations, extremely high computational cost and overall very simplified reaction kinetics. This limited the predictive capability of the model to be extended to different aggregate types and it only replicates SEM images of the samples that were tested. However, it was able to demonstrate that in many cases, there is no need to assume diffusion of the produced gel from the silica pockets to replicate damage and only pocket expansion is enough.

Generally, the length scale at which computational models are developed allows capturing the phenomena at that length scale and requires that lower scale phenomena are averaged and included in the constitutive equations. The choice of the length scale is a challenge where a trade off must be considered between the model accuracy and maximum size of structure that can be eventually be simulated. For example, as appealing molecular dynamics simulations can be, they are usually limited to the simulation of volumes of material in the nanometer range. Therefore, there is no chance at the moment to use these approaches to model even one single aggregate piece.

In 2013, Alnaggar et al. [65] proposed a multiscale model for ASR deterioration of concrete structures entitled ASR-LDPM and simulating ASR effects within the Lattice Discrete Particle Model (LDPM) [66,67]. LDPM, in a full 3D setting, simulates the mechanical interaction of coarse aggregate pieces through a system of three-dimensional polyhedral particles, each resembling a spherical coarse aggregate piece with its surrounding mortar, connected through lattice struts [66] and it has the ability of simulating the effect of material heterogeneity on the fracture process [67]. ASR-LDPM unprecedentedly replicated all general aspects of ASR expansion and its degradation effects without the need of postulating any phenomenological assumptions between stress state or material constitutive behavior and the ASR kinetics. Such a unique feature was made possible by distinguishing between ASR gel and ASR induced cracking, two sources of measured expansion that have been always denoted by other researchers as a combined ASR strain. The model was limited to fully saturated conditions and accounted for the accompanying shrinkage and creep strains at the macroscopic level only. ASR-LDPM was able to explain the experimental results of non-destructive testing of concrete using ultrasonic techniques [68]. The model replicated the change in acoustic nonlinearity parameter and correlated it to the cracking volume and pattern. Till today, there has been no other model able to replicate these measurements in concrete without any explicit introduction of constitutive relationships relating expansion to damage. The model also was extended to consider alkali nonlinear diffusion and proposed a concentration dependent diffusivity parameter for alkali that accounts for the effect of concrete pore charge on diffusivity [68]. In addition, in his PhD work, Alnaggar [9] also proposed an extension of the ASR-LDPM model to unsaturated conditions and its coupling with creep and shrinkage deformations.

More recently, other multi-scale models appeared in the literature. Multon et al. modeled the effect of alkali leaching by modeling alkali macro-diffusion and its effects on expansion of specimens [69]. While the model did include leaching effects, the simplified assumption of linear diffusion represent a contradiction with available literature data on alkali diffusion [70,71,72]. In addition, the phenomenological damage mechanics constitutive formulation of the model reduces also its capability to realistically capture all ASR expansion and damage aspects, not to mention its coupling with other phenomena. An extended version of the model was used to simulate both ASR and DEF expansions considering their coupling with leaching and moisture transfer. The model and accompanying experiments showed the importance of such coupling especially for large crack openings [73]. Another comprehensive model considering possible migration of ASR gel and its diffusion within the concrete porous structure was recently presented by Bažant et al. [10]. This model also considered creep effects, stress effects and humidity effects on ASR expansion and the resulting damage [74].

In the present study, ASR-LDPM [65] is reviewed and extended to variable moisture conditions and is implemented within a multi-scale multi-physics framework that takes into consideration spatial and temporal distributions of humidity and temperature inside concrete. All accompanying deformations, such as shrinkage, thermal and creep strains, are also introduced in the numerical framework allowing not only the macroscopic effects of those deformations but notably the effects of creep-induced stress relaxation of ASR induced internal pressure. The humidity, temperature and cement hydration calculations are performed using an FE multi-physics framework and are interpolated at the facets of the mechanical model (LDPM).

With the exception of the Microplane model [10], macroscopic homogeneous continuum models can not simulate such feature. This is because, in case of free expansion, all ASR stresses are self equilibrated and don’t contribute to the macroscopic stress state, thus the macroscopic stress tensor is zero and that does not produce any creep strain. This can be seen for example in the model by Kawabata et al. [2], which while successful in many regards, it considers macroscopic creep only.

## 2. Multi-Physics Formulation

### 2.1. Modeling ASR Expansion

Considering the observations on aggregate nature, its chemistry, and the chemistry of the surrounding binder mentioned in Section 1, it is obvious that, regardless of the sub-scale phenomena that govern the ASR expansion and cracking within the aggregate, a diffusion process at the meso-scale has to happen to transport alkali ions into the aggregate for ASR gel to form and to also transport water for the gel to imbibe and later expand. Considering also, the non-feasible simulation of full 3D structural elements using micro-to nano-scale models, in this study the choice was made to use a meso-scale model (ASR-LDPM) [65,68,75], that would be capable of reflecting the major phenomena at that length scale and average sub-scale ones. ASR-LDPM implements, within the mesoscale framework of LDPM, a model describing ASR gel formation and expansion at the level of each individual aggregate particle. The overall average rate of expansion of a single aggregate piece is related to two main processes: (1) basic gel formation; and (2) water imbibition. Subsequently, the volume increase due to water imbibition is imposed as an eigenstrain within the LDPM model.

**Figure 1:**
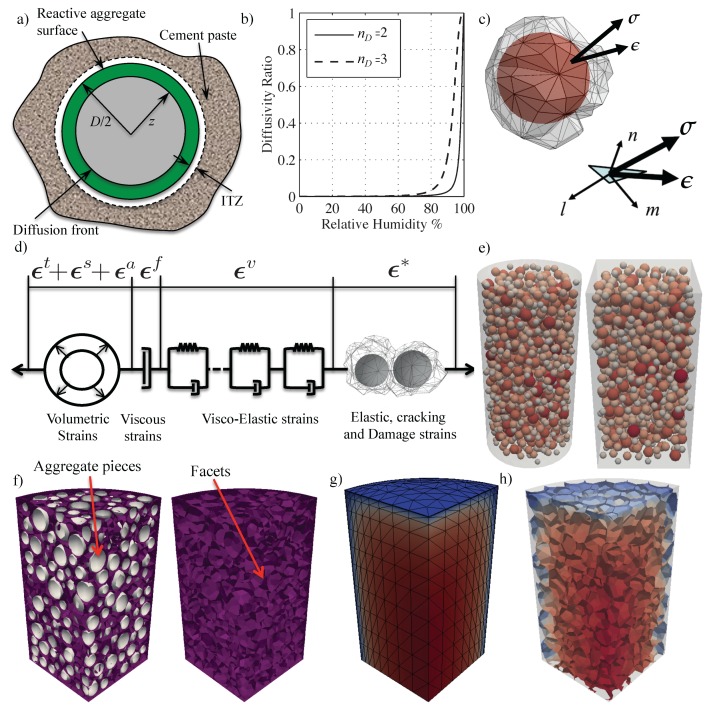
(**a**) Idealization of gel formation in one aggregate; (**b**) Diffusivity change with relative humidity; (**c**) One Lattice Discrete Particle Model (LDPM) Cell around an aggregate piece; (**d**) Equivalent rheological model based on strain additivity; (**e**) Cylinder and Prism generated LDPM geometries (Aggregate are colored by their relative size); (**f**) 1/8th of the simulated cylinder showing the discrete facets inside it surrounding the aggregate; (**g**) 1/8th Hygro-Thermo-Chemical (HTC) cylindrical mesh colored by the values from RH field for the drying case at 420 days; (**h**) The interpolated values of RH from the HTC mesh into LDPM facets centroids.

*Gel Formation.* Similarly to the work in Reference [65], the formulation focuses on the mesoscale (length scale of coarse aggregate pieces) and finer scale mechanisms are accounted for in an average sense through mesoscale governing equations. The gel mass $M_{g}$ generated from an aggregate particle with diameter *D*, is derived by solving the equation governing a radial diffusion process into the aggregate particle (see Figure 1a). This is justified by the fact that, regardless of the fine scale characteristics of gel formation, water and alkali ions must diffuse through aggregate particle to reach the silica. Thus, one can write,

(1) $$M_{g}=\kappa_{a}\rho_{g}\frac{\pi }{6}\left(D^{3}-8z^{3}\right)$$

and

(2) $$\dot{z}=-\kappa_{z}\frac{w_{e}}{z\left(1-\frac{2z}{D}\right)}$$

where $w_{e}=$ water content in the concrete surrounding the aggregate particle estimated based on Reference [76] as $w_{e}=\left(w/c-0.188\alpha_{c}^{\infty }\right)c$ at saturation; $\alpha_{c}^{\infty }=\left(1.031\;w/c\right)/\left(0.194+w/c\right)$ asymptotic hydration degree [77]; w/c= water-to-cement ratio; c= cement content. Furthermore, in Equations (1) and (2), z= the diffusion front position measured from each aggregate particle center as shown in Figure 1a, $\rho_{g}$ (with units kg/m${}^{3}$) represents the mass density of gel formed and it depends on its chemical composition and silica content per aggregate volume. $\kappa_{z}=\kappa_{z0}\exp\left(\frac{E_{ag}}{RT_{0}}-\frac{E_{ag}}{RT}\right)$ represents the diffusivity of alkali rich water into the aggregate and has units of m${}^{5}/$(kg day) where $\kappa_{z0}$ is its value at room temperature ($T_{0}=296$${}^{\circ }K$); T= current temperature; $E_{ag}=$ activation energy of the diffusion process; and R= universal gas constant. $\kappa_{a}=\min\left(\langle c_{a}-c_{a0}\rangle /\left(c_{a1}-c_{a0}\right),1\right)$ accounts for the fact that alkali content available in the cement paste surrounding each aggregate particle, is not always enough for the ASR reaction to occur; $c_{a0}$ is the threshold alkali content at which, no or minimal expansion is observed, and $c_{a1}$ is the saturation alkali content enough for complete silica reaction. Note that *z* might represent, depending on the situation, the evolution of different phenomena, from the thickness of the reaction rim to the extent of the penetration of alkali rich water needed for the reaction of isolated silica pockets.

*Water Imbibition.* The water imbibition process is described by relating the rate of water mass $M_{i}$ imbibed by gel to the thermodynamic affinity and a characteristic imbibition time. Considering the gel mass $M_{g}$ given by the integration of Equation (2), the rate of water imbibition is given by:

(3) $${\dot{M}}_{i}=\frac{C_{i}}{\delta^{2}}\left[\kappa_{i}M_{g}-M_{i}\right]$$

where the imbibed water at thermodynamic equilibrium has been assumed to be linearly proportional to the mass of formed gel with $\kappa_{i}=\kappa_{i0}\exp\left(\frac{E_{ai}}{RT_{0}}-\frac{E_{ai}}{RT}\right)$ as the constant of proportionality, and temperature-dependent through an Arrhenius-type equation governed by the activation energy of the imbibition capacity, $E_{ai}$, and is its value at room temperature, $\kappa_{i0}$. Similarly, $C_{i}=C_{i0}\exp\left(\frac{E_{aw}}{RT_{0}}-\frac{E_{aw}}{RT}\right)$ represents the bulk diffusivity of imbibed water through both the cement paste surrounding the aggregate and the reacted external rim of the aggregate; $E_{aw}$ = diffusion process activation energy and $C_{i0}$ = value at room temperature. δ is the average (or effective) distance of water transport process from the concrete around the aggregate into the ASR gel. Similarly to Reference [10], it is reasonable to assume that δ is proportional to the aggregate diameter *D* as $\delta =\alpha_{M}D$ where $\alpha_{M}$ is a small fraction and can be assumed to be about 1%. With this assumption, an effective diffusivity parameter ${\tilde{C}}_{i}=C_{i}/\alpha_{M}^{2}$ can be defined and thus the rate of water imbibition can be re-written as:

(4) $${\dot{M}}_{i}=\frac{{\tilde{C}}_{i}}{D^{2}}\left[\kappa_{i}M_{g}-M_{i}\right]$$

The inverse of the ratio $C_{i}/\delta^{2}={\tilde{C}}_{i}/D^{2}$ here represents the imbibition rate characteristic time as was explained earlier in References [3,65]. The characteristic time is assumed to be constant at full saturation, but depending on silica distribution, type of aggregate, porosity and the inter-connectivity and tortuosity of its pore system, this coefficient can vary with the amount of imbibed water. Two competing factors are expected to affect the characteristic time, the first is the increase of water transport path as the diffusion front advances along with the possible clogging of pores due to water imbibition. This would result in longer characteristic time. The other is that as the reaction advances, the aggregate cracks and cracks can easily increase the diffusivity resulting in a smaller characteristic time. So, with the limited information at hand and due to the vast variability of the aggregate sources and other aforementioned factors, the simplified constant assumption seems reasonable [3,65].

*Extension for nonsaturated conditions.* In real situations, structures are not fully saturated and a variable distribution of humidity over the cross-section or along the length of the structural element is generally possible. The amount of moisture content is typically governed by a nonlinear diffusion process with a nonlinear temperature dependence which, in turn, considerably affect ASR generation and imbibition [65]. ASR-LDPM is extended here to account for nonsaturated conditions. The spatial and temporal distributions of relative humidity *h*, temperature *T* and degree of cement hydration $\alpha_{c}$ are computed using the Hygro-Thermo-Chemical (HTC) model (described in the next section). The first step is to account for the amount of evaporable water, $w_{e}$, in the surrounding of aggregate particles which depends on the relative humidity in the pores and the aging of the cement paste. According to Reference [76] one can write:

(5) $$w_{e}\left(h,\alpha_{c}\right)=G_{1}\left(\alpha_{c}\right)\left[1-\frac{1}{e^{10(g_{1}\alpha_{c}^{\infty }-\alpha_{c})h}}\right]+K_{1}\left(\alpha_{c}\right)\left[e^{10(g_{1}\alpha_{c}^{\infty }-\alpha_{c})h}-1\right]$$

(6) $$G_{1}\left(\alpha_{c}\right)=g_{2}\alpha_{c}c$$

(7) $$K_{1}\left(\alpha_{c}\right)=\frac{w_{0}-0.188\alpha_{c}cG_{1}\left(\alpha_{c}\right)\left[1-e^{-10(g_{1}\alpha_{c}^{\infty }-\alpha_{c})}\right]}{e^{10(g_{1}\alpha_{c}^{\infty }-\alpha_{c})}-1}$$

where $g_{1}$ and $g_{2}$ are material parameters. Equation (5) does not account for the water consumed in the ASR process. This is a reasonable assumption because the cement hydration process varies significantly only within the first months of concrete life and humidity variations are usually within seasonal cycles (at least at concrete surface) while the ASR process is a multi-decade process. This means that the time scales that contribute to $w_{e}$ variations are different and typical variations due to relative humidity and aging are time sub-scales of the ASR process. Similar observations apply to variations of relative humidity and temperature. In other words, in this study, there is only one way coupling between the hygro-thermo-chemical processes and the ASR process. All field variables (*h*, *T* and $\alpha_{c}$) are calculated according to the HTC model [76] (reviewed later in Section 2.2) assuming no effects from ASR evolution. For nonsaturated humidity environments, the imbibition is dramatically reduced, and at relative humidity lower than 60%–80%, no noticeable expansions are reported [1]. The effect of relative humidity is introduced into the diffusion front speed $\dot{z}$ by making the diffusivity parameter $\kappa_{z}$ a function of *h* as:

(8) $$\kappa_{z}\left(h,T\right)=\kappa_{z}^{1}{\left[1+\left(\frac{\kappa_{z}^{1}}{\kappa_{z}^{0}}-1\right){\left(1-h\right)}^{n_{Z}}\right]}^{-1}$$

where $\kappa_{z}^{1}=\kappa_{z0}^{1}\exp\left(\frac{E_{ag}}{RT_{0}}-\frac{E_{ag}}{RT}\right)$ diffusivity at the current temperature *T* and full saturation (h=1); $\kappa_{z0}^{1}=$ diffusivity at room temperature $T_{0}$ and full saturation (h=1); $\kappa_{z}^{0}=\kappa_{z0}^{0}\exp\left(\frac{E_{ag}}{RT_{0}}-\frac{E_{ag}}{RT}\right)$ diffusivity at the current temperature *T* and dry condition (h=0); $\kappa_{z0}^{0}=$ diffusivity at room temperature $T_{0}$ and dry condition (h=0); $n_{Z}$ is a model parameter. This changes the diffusion front speed Equation (2) into:

(9) $$\dot{z}=-\kappa_{z}\left(h,T\right)\frac{w_{e}\left(h,\alpha_{c}\right)}{z\left(1-\frac{2z}{D}\right)}$$

The ASR gel water imbibition is also affected by the relative humidity. It is reasonable to account for this additional effect by postulating a dependence of the effective diffusivity parameter ${\tilde{C}}_{i}$ on the relative humidity *h*. This is captured by setting:

(10) $${\tilde{C}}_{i}\left(h,T\right)={\tilde{C}}_{i}^{1}{\left[1+\left(\frac{{\tilde{C}}_{i}^{1}}{{\tilde{C}}_{i}^{0}}-1\right){\left(1-h\right)}^{n_{M}}\right]}^{-1}$$

where ${\tilde{C}}_{i}^{1}={\tilde{C}}_{i0}^{1}\exp\left(\frac{E_{aw}}{RT_{0}}-\frac{E_{aw}}{RT}\right)$ is the effective diffusivity at full saturation (h=1) and the current temperature *T*; ${\tilde{C}}_{i0}^{1}$ is the effective diffusivity at full saturation (h=1) and room temperature $T_{0}$; ${\tilde{C}}_{i}^{0}={\tilde{C}}_{i0}^{0}\exp\left(\frac{E_{aw}}{RT_{0}}-\frac{E_{aw}}{RT}\right)$ is the effective diffusivity at dry condition (h=0) and the current temperature *T*; ${\tilde{C}}_{i0}^{0}$ is the effective diffusivity at dry condition (h=0) and room temperature $T_{0}$; and $n_{M}$ is a model parameter.

By considering all these effects together and taking into account Equation (8), the governing equation for water imbibition into the gel previously given by Equation (4) becomes:

(11) $${\dot{M}}_{i}=\frac{{\tilde{C}}_{i}\left(h,T\right)}{D^{2}}\left[\kappa_{i}M_{g}-M_{i}\right]$$

The assumed functional forms of both $\kappa_{z}\left(h,T\right)$ and ${\tilde{C}}_{i}\left(h,T\right)$ are essentially inherited from the moisture permeability dependence on *h* as presented in Reference [76] and reported later in Equation (14). This is mainly because, we assume that the rate of both processes is controlled by moisture diffusion. Although one can argue that the formation of basic gel requires the diffusion of alkali, we assume, as confirmed by physical observations, that the amount of alkali transported by convection (through water movement) dominate compared to the one carried by molecular diffusion through the solid structure of the aggregate. Finally, in absence of specific experimental data, it is assumed that, at constant temperature, the ratio of gel diffusivities is equal to the ratio of water imbibition diffusivities $\kappa_{z}^{1}/\kappa_{z}^{0}={\tilde{C}}_{i}^{1}/{\tilde{C}}_{i}^{0}=r_{D}$ and also the exponents are the same $n_{z}=n_{M}=n_{D}$. This is basically equivalent to assuming that the effects of relative humidity variations are the same for both processes. The dependence on *h* is plotted for two different exponents ($n_{D}=2$ and $n_{D}=3$) in Figure 1b and it shows that the adopted functional form is consistent with almost complete suppression of ASR evolution for relative humidity levels smaller than 0.8. This approach was first introduced by Alnaggar [9] and then was also utilized by Bažant et al.

### 2.2. Hygro-Thermo-Chemical (HTC) Model

To be able to describe the interaction and coupling between various aging and deterioration phenomena along with changes in environmental conditions, the values of temperature, *T*, relative humidity, *h*, and cement hydration degree, $\alpha_{c}$, must be spatially and temporally defined over the structural element with enough resolution so that their differences around aggregate pieces are captured. This is essential for capturing creep and shrinkage deformations in a meso-scale setting. In addition, as previously discussed, ASR processes are strongly dependent on temperature and humidity. This means that rough average measures of any of these variables are not enough to properly describe the ASR evolution and thus, the need for precise reliable modeling of the moisture and temperature transport and distribution within the concrete internal structure becomes essential. A comprehensive three dimensional Hygro-Thermo-Chemical (HTC) model [76] for the evolution of temperature, humidity and cement hydration degree is adopted in this study. Based on this model, *h* and *T* distributions can be computed by imposing moisture mass balance and enthalpy balance equations in the volume of interest. For concrete mixes in which the binder is Portland cement and for temperature not exceeding $90{\;}^{\circ }$C, one can write [76]

(12) $$\nabla \cdot \left(D_{h}\nabla h\right)-\frac{\partial w_{e}}{\partial h}\frac{\partial h}{\partial t}-\frac{\partial w_{e}}{\partial \alpha_{c}}{\dot{\alpha }}_{c}-{\dot{w}}_{n}=0$$

and

(13) $$\nabla \cdot \left(\lambda_{t}\nabla T\right)-\rho c_{t}\frac{\partial T}{\partial t}+\dot{\alpha_{c}}\;c\;{\tilde{Q}}_{c}^{\infty }=0$$

where c= cement content, $D_{h}$ is moisture permeability, $w_{e}$ is evaporable water, $\alpha_{c}=$ hydration degree, ${\dot{w}}_{n}=0.253{\dot{\alpha }}_{c}c$ is rate of non-evaporable water, ρ= mass density of concrete, $c_{t}=$ isobaric heat capacity (specific heat), $\lambda_{t}=$ heat conductivity, ${\tilde{Q}}_{c}^{\infty }=$ hydration enthalpy. Typically ${\tilde{Q}}_{c}^{\infty }\approx 450$ kJ/kg. Note here that in both Equations (12) and (13) there are no sink terms for water consumption nor heat consumption/generation by ASR consistently with what was discussed previously.

The moisture permeability is assumed to be a nonlinear function of the relative humidity *h* and temperature *T*, and is formulated as follows

(14) $$D_{h}\left(h,T\right)=\exp\left(\frac{E_{ad}}{RT_{0}}-\frac{E_{ad}}{RT}\right)D_{1}{\left[1+\left(\frac{D_{1}}{D_{0}}-1\right){\left(1-h\right)}^{n}\right]}^{-1}$$

where $T_{0}=296$${}^{\circ }$K, $E_{ad}/R\approx 2700$ K.

The evaporable water (sorption/desorption isotherm) can be assumed to be a function of relative humidity and degree of hydration [78] and it is formulated through Equations (5)–(7).

Diluzio and Cusatis [79] report detailed calibration and validation of this theory. It must be noted here that this theory does not account, as first approximation, for typically observed hysteresis during adsorption/desorption cycles [80,81], which has been recently explained by Bažant and Bažant [82] to be the consequence of two related mechanisms: snap-through instabilities during the filling or emptying of non-uniform nanopores or nanoscale asperities and the molecular coalescence, or capillary condensation, within a partially filled surface. In addition, the moisture permeability defined in Equation (14) does not account for the cracking effect due to ASR. This approximation is relatively valid here as the modeled experimental expansions are small. But when expansions are larger (either due to ASR or other phenomena like DEF) the cracking effect on permeability and leaching becomes very important as it has been shown by Martin et al. [73] and Kawabata et al. [83].

For the concrete mixes of interest in this study, the main early-age chemical reaction is the cement hydration—the reaction of free-water with unhydrated cement particles. This reaction generates Calcium-Silicate-Hydrates (C-S-H) which is the main constituent providing stiffness and strength to concrete.

Cement hydration can be characterized by the hydration degree [76,84,85,86], $\alpha_{c}$, that represents the fraction of Portland clinker fully reacted with water. Its evolution law can be formulated as

(15) $${\dot{\alpha }}_{c}=\frac{A_{c1}e^{-\eta_{c}\alpha_{c}/\alpha_{c}^{\infty }}e^{-E_{ac}/R\left(T-T_{0}\right)}}{1+{\left(5.5-5.5h\right)}^{4}}\left(\frac{A_{c2}}{\alpha_{c}^{\infty }}+\alpha_{c}\right)\left(\alpha_{c}^{\infty }-\alpha_{c}\right)$$

where $E_{ac}/R\approx 5000{/}^{\circ }$K, $T_{0}=296^{\circ }$K and $\eta_{c}$, $A_{c1}$, $A_{c2}$ are material parameters.

### 2.3. Mechanical Behavior

In this research, ASR induced deformations, in addition to thermal, shrinkage and creep deformations are formulated within the framework of the Lattice Discrete Particle Model (LDPM).

The Lattice Discrete Particle Model (LDPM) [66,67] is a meso-scale discrete model that simulates the mechanical interaction of coarse aggregate pieces embedded in a cementitious matrix (mortar). The geometrical representation of concrete mesostructure is constructed through the following steps. (1) The coarse aggregate pieces, whose shapes are assumed to be spherical, are introduced into the concrete volume by a try-and-reject random procedure. (2) Zero-radius aggregate pieces (nodes) are randomly distributed over the external surfaces to facilitate the application of boundary conditions. (3) A three-dimensional domain tessellation, based on the Delaunay tetrahedralization of the generated aggregate centers, creates a system of polyhedral cells interacting through triangular facets and a lattice system composed by the line segments connecting the particle centers. Figure 1c shows an idealized spherical aggregate piece surrounded by the generated system of interaction facets. The two vectors shown in Figure 1c are the stress vector σ and strain vector ϵ acting on this facet. The equation of motion (in absence of body forces) of a generic LDPM cell reads:

(16) $$\sum_{F}A\sigma =m_{u}\ddot{u}\;;\;\sum_{F}Ac\times \sigma =m_{\omega }\ddot{\omega }$$

where F is the set of facets defining the cell, *A* is each facet area, $m_{u}$ is the mass of the cell, $m_{\omega }$ is the rotational inertia of the cell, and $\ddot{u},\ddot{\omega }$ are acceleration and rotational acceleration, respectively, of the cell center.

The stress vector $\sigma ={\left[t_{N}\;t_{M}\;t_{L}\right]}^{T}$ is assumed to be uniform over each facet and is computed through constitutive relationships, σ=f(ϵ), governing the behavior of the material.

In LDPM, rigid body kinematics is used to describe the deformation of the lattice/particle system and the displacement jump, $〚u_{C}〛$, at the centroid of each facet is used to define measures of strain as

(17) $$e_{N}=\frac{n^{T}〚u_{C}〛}{\ell };\;\;\;e_{L}=\frac{l^{T}〚u_{C}〛}{\ell };\;\;\;e_{M}=\frac{m^{T}〚u_{C}〛}{\ell }$$

where ℓ= interparticle distance; and n, l, and m, are unit vectors defining a local system of reference attached to each facet, and $\epsilon ={\left[e_{N}\;e_{M}\;e_{L}\right]}^{T}$ represents the facet material strain vector (see Figure 1c). It was recently demonstrated that the strain definitions in Equation (17) correspond to the projection into the local system of references of the strain tensor typical of continuum mechanics [87,88,89]. By assuming additivity of strains, one can write:

(18) $$\dot{\epsilon }={\dot{\epsilon }}^{*}+{\dot{\epsilon }}^{a}+{\dot{\epsilon }}^{s}+{\dot{\epsilon }}^{t}+{\dot{\epsilon }}^{v}+{\dot{\epsilon }}^{f}$$

where ${\dot{\epsilon }}^{*}$ represents the effect of instantaneous elasticity and damage, ${\dot{\epsilon }}^{a}$ represents the ASR induced strain rate; ${\dot{\epsilon }}^{s}$ and ${\dot{\epsilon }}^{t}$ are shrinkage and thermal strain rates (respectively); ${\dot{\epsilon }}^{v}$ is the viscoelastic strain rate and ${\dot{\epsilon }}^{f}$ is the purely viscous strain rate. Equation (18) can be seen as the mathematical interpretation of the rheological model depicted in Figure 1d.

#### 2.3.1. LDPM for Concrete Elastic, Cracking and Damage Behavior

In the elastic regime, the normal and shear stresses are proportional to the corresponding strains: $t_{N}=E_{N}e_{N}^{*};\;t_{M}=E_{T}e_{M}^{*};\;t_{L}=E_{T}e_{L}^{*}$, where $E_{N}=E_{0}$, $E_{T}=\alpha E_{0}$, $E_{0}=$ effective normal modulus, and α= shear-normal coupling parameter. In vectorial form, one has $\epsilon^{*}=1/E_{0}G\sigma$ where:

(19) $$G=\left[\begin{array}{ccc} 1 & 0 & 0\\ 0 & 1/\alpha & 0\\ 0 & 0 & 1/\alpha \end{array}\right]$$

It must be observed here that in theory, $E_{0}$ should not account for any creep deformation that always occurs during quasi-static tests because all creep strains are included in the Kelvin chain of the rheological model. In practice, however, the Kelvin chain is always approximated by a finite chain and, in this case, $E_{0}$ will also include the effect of very short term creep whose characteristic time is smaller than the smallest of the discrete chain. More discussion of this point is reported in Section 4.

*Fracture and cohesion due to tension and tension-shear.* For tensile loading ($e_{N}^{*}>0$), the fracturing behavior is formulated through an effective strain, $e=\sqrt{e_{N}^{*2}+\alpha \left(e_{M}^{*2}+e_{L}^{*2}\right)}$, and stress, $t=\sqrt{t_{N}^{2}+\left(t_{M}^{2}+t_{L}^{2}\right)/\alpha }$, which define the normal and shear stresses as $t_{N}=e_{N}^{*}\left(t/e\right)$; $t_{M}=\alpha e_{M}^{*}\left(t/e\right)$; $t_{L}=\alpha e_{L}^{*}\left(t/e\right)$. The effective stress *t* is incrementally elastic ($\dot{t}=E_{0}\dot{e}$) and must satisfy the inequality $0\leq t\leq \sigma_{bt}\left(e,\omega \right)$ where $\sigma_{bt}=\sigma_{0}\left(\omega \right)\exp\left[-H_{0}\left(\omega \right)\langle e-e_{0}\left(\omega \right)\rangle /\sigma_{0}\left(\omega \right)\right]$, 〈x〉=max{x,0}, and $\tan\left(\omega \right)=e_{N}^{*}/\sqrt{\alpha }e_{T}^{*}$ = $t_{N}\sqrt{\alpha }/t_{T}$, and $e_{T}^{*}=\sqrt{e_{M}^{*2}+e_{L}^{*2}}$. The post peak softening modulus is defined as $H_{0}\left(\omega \right)=H_{t}{\left(2\omega /\pi \right)}^{n_{t}}$, where $H_{t}$ is the softening modulus in pure tension (ω=π/2) expressed as $H_{t}=2E_{0}/\left(\ell_{t}/\ell -1\right)$; $\ell_{t}=2E_{0}G_{t}/\sigma_{t}^{2}$; *ℓ* is the length of the tetrahedron edge; and $G_{t}$ is the mesoscale fracture energy. LDPM provides a smooth transition between pure tension and pure shear (ω=0) with parabolic variation for strength given by $\sigma_{0}\left(\omega \right)=\sigma_{t}r_{st}^{2}(-\sin\left(\omega \right)+\;\sqrt{\sin^{2}\left(\omega \right)+4\alpha \cos^{2}\left(\omega \right)/r_{st}^{2}})/\left[2\alpha \cos^{2}\left(\omega \right)\right]$, where $r_{st}=\sigma_{s}/\sigma_{t}$ is the ratio of shear strength to tensile strength.

*Compaction and pore collapse in compression.* To simulate pore collapse and material compaction, LDPM normal stresses for compressive loading ($e_{N}^{*}<0$) are computed through the inequality $-\sigma_{bc}\left(e_{D}^{*},e_{V}^{*}\right)\leq t_{N}\leq 0$, where $\sigma_{bc}$ is a strain-hardening boundary assumed to be a function of the volumetric strain, $e_{V}^{*}$, and the deviatoric strain, $e_{D}^{*}=e_{N}^{*}-e_{V}^{*}$. The volumetric strain is computed by the volume variation of the Delaunay tetrahedra as $e_{V}^{*}=\Delta V/3V_{0}$ and is assumed to be constant for all facets belonging to a given tetrahedron. Beyond the elastic limit, $-\sigma_{bc}$ is defined as : $-\sigma_{bc}\left(e_{D}^{*},e_{V}^{*}\right)=\sigma_{c0}$ for $-e_{DV}^{*}\leq 0$, $-\sigma_{bc}\left(e_{D}^{*},e_{V}^{*}\right)=\sigma_{c0}+\langle -e_{DV}^{*}-e_{c0}\rangle H_{c}\left(r_{DV}\right)$ for $0\leq -e_{DV}^{*}\leq e_{c0}$, and $-\sigma_{bc}\left(e_{D}^{*},e_{V}^{*}\right)=\sigma_{c1}\left(r_{DV}\right)$$\exp\left[\left(-e_{DV}^{*}-e_{c1}\right)H_{c}\left(r_{DV}\right)/\sigma_{c1}\left(r_{DV}\right)\right]$ otherwise. Where $e_{DV}^{*}=e_{V}^{*}+\beta e_{D}^{*}$, β is a material parameter, $\sigma_{c0}$ is the mesoscale compressive yield stress, $e_{c0}=\sigma_{c0}/E_{0}$ is the compaction strain at the beginning of pore collapse, $H_{c}\left(r_{DV}\right)$ is the hardening modulus, $e_{c1}=\kappa_{c0}e_{c0}$ is the compaction strain at which rehardening begins, $\kappa_{c0}$ is the material parameter governing the rehardening and $\sigma_{c1}\left(r_{DV}\right)=\sigma_{c0}+\left(e_{c1}-e_{c0}\right)H_{c}\left(r_{DV}\right)$. In Ceccato et al. [90], the hardening modulus is given by $H_{c}\left(r_{DV}\right)=H_{c1}+\left(H_{c0}-H_{c1}\right)/\left(1+\kappa_{c2}\langle r_{DV}-\kappa_{c1}\rangle \right)$, with $r_{DV}=\left|e_{D}^{*}\right|/\left(e_{V0}-e_{V}^{*}\right)$ for $e_{V}^{*}\leq 0$ and $r_{DV}=\left|e_{D}^{*}\right|/e_{V0}$ for $e_{V}^{*}>0$, $e_{V0}=0.1e_{c0}$, $\kappa_{c1}=1$, $\kappa_{c2}=5$ and $H_{c0}$, $H_{c1}$ are assumed to be material parameters.

*Friction due to compression-shear.* The incremental shear stresses are computed as ${\dot{t}}_{M}=E_{T}\left({\dot{e}}_{M}^{*}-{\dot{e}}_{M}^{*p}\right)$ and ${\dot{t}}_{L}=E_{T}\left({\dot{e}}_{L}^{*}-{\dot{e}}_{L}^{*p}\right)$, where ${\dot{e}}_{M}^{*p}=\dot{\lambda }\partial \varphi /\partial t_{M}$, ${\dot{e}}_{L}^{*p}=\dot{\lambda }\partial \varphi /\partial t_{L}$, and λ is the plastic multiplier with loading-unloading conditions $\varphi \dot{\lambda }\leq 0$ and $\dot{\lambda }\geq 0$. The plastic potential is defined as $\varphi =\sqrt{t_{M}^{2}+t_{L}^{2}}-\sigma_{bs}\left(t_{N}\right)$, where the nonlinear frictional law for the shear strength is assumed to be $\sigma_{bs}=\sigma_{s}+\left(\mu_{0}-\mu_{\infty }\right)\sigma_{N0}\left[1-\exp\left(t_{N}/\sigma_{N0}\right)\right]-\mu_{\infty }t_{N}$; $\sigma_{N0}$ is the transitional normal stress; $\mu_{0}$ and $\mu_{\infty }=0$ are the initial and final internal friction coefficients.

LDPM has been used successfully to simulate concrete behavior under a large variety of loading conditions [66,67]. Furthermore it can be properly formulated to account for fiber reinforcement [91,92] and it was recently extended to simulate the ballistic behavior of ultra-high performance concrete (UHPC) [93]. In addition, LDPM was successfully used in structural element scale analysis using multiscale methods [88,94,95] and was also used to simulate compression failure of confined concrete columns with FRP wrapping [90].

### 2.4. Microprestress-Solidification Theory for Viscous and Visco-Elastic Deformations

According to the Microprestress Solidification Theory [96,97,98], the visco-elastic behavior of concrete is modeled through the sum of two strain components: the visco-elastic strain and the purely viscous strain.

The viscoelastic strain rate is formulated as:

(20) $${\dot{\epsilon }}^{v}\left(t\right)=\frac{1}{v(\alpha_{c})}\dot{\gamma };\;\;\;\;\;\gamma =\int_{0}^{t}\Phi \left(t_{r}\left(t\right)-t_{r}\left(\tau \right)\right)G\dot{\sigma }d\tau$$

where $\dot{\gamma }$ represents the cement gel viscoelastic micro-strain rate, $v\left(\alpha_{c}\right)={\left(\alpha_{c}/\alpha_{c}^{\infty }\right)}^{n_{\alpha }}$ is a function that represents the volume fraction of cement gel produced by early-age chemical reactions, $\Phi \left(t-t_{0}\right)=\xi_{1}\ln\left[1+{\left(t-t_{0}\right)}^{0.1}\right]$ is the non-aging micro-compliance function of cement gel, with $t-t_{0}$ as the loading time interval. $\xi_{1}$ and $n_{\alpha }$ are model parameters. To account for the effect of change in relative humidity and temperature the reduced time concept is used [99], where $t_{r}\left(t\right)=\int_{0}^{t}\psi \left(\tau \right)d\tau$ and $\psi \left(t\right)=\left[0.1+0.9h^{2}\right]\exp\left[Q_{v}/R\left(1/T_{0}-1/T\right)\right]$, where *h*, *T* are the relative humidity and temperature (in Kelvin) at time *t*, *R* is the universal gas constant and $Q_{v}$ is the activation energy for the creep processes. For typical concrete mixes $Q_{v}/R\approx$ 5000 K [96].

The purely viscous strain rate represents the totally unrecoverable part of the creep strain and it is associated to long-term creep, drying creep effect (also called Pickett effect) and transitional thermal creep. One can write:

(21) $${\dot{\epsilon }}^{f}=\xi_{2}\kappa_{0}\psi \left(t\right)SG\sigma$$

where *S* is the microprestress computed by solving the differential equation $\dot{S}+\psi_{s}\left(t\right)\kappa_{0}S^{2}=\kappa_{1}\left|\dot{T}\ln\left(h\right)+T\dot{h}/h\right|$, where $\kappa_{0}$, $\kappa_{1}$ and $\xi_{2}$ are model parameters. Furthermore, $\psi_{s}\left(t\right)=\left[0.1+0.9h{\left(t\right)}^{2}\right]\exp\left[Q_{s}/R\left(1/T_{0}-1/T\left(t\right)\right)\right]$ and, typically, $Q_{s}/R\approx$ 3000 K [96]. In this differential equation, the initial value $S_{0}$ at time $t=t_{0}$ must be defined and it is assumed to be a model parameter [99]. However, if one assumes, as verified by experiments, that the purely viscous strain is a logarithmic function of time in the case of basic creep, one has $S_{0}\kappa_{0}t_{0}=1$ where $t_{0}=1$ day can be assumed without loss of generality. It must be observed here that, the three parameters, $\kappa_{0},\kappa_{1},\xi_{2}$ are not independent as far as the viscous strain is concerned. Basic creep viscous strain depends on $\xi_{2}$ only [99]; drying and transitional thermal creep depend on $\xi_{2}$ and the product $\kappa_{0}\kappa_{1}$ [100] This is simple to show by introducing the auxiliary variable $\bar{S}=\kappa_{0}S$. One has ${\dot{\epsilon }}^{f}=\xi_{2}\bar{S}\psi \left(t\right)G\sigma$, $\dot{\bar{S}}+\psi_{s}\left(t\right){\bar{S}}^{2}=\kappa_{0}\kappa_{1}\left|\dot{T}\ln\left(h\right)+T\dot{h}/h\right|$ [101]. Hence, the value of $\kappa_{0}=$ 2×10${}^{-3}$ MPa/day will be used in this paper. Independent identification of $\kappa_{0}$ requires experimental data on the microprestress evolution. Such data is not available at the moment.

### 2.5. ASR Induced Deformation

The water imbibition rate ${\dot{M}}_{i}$ for a specific aggregate piece is given by Equation (11). If there is no room for the additional mass to be accommodated, the aggregate starts to swell. In many cases, initial expansion of the ASR gel can be partly accommodated without significant pressure build up by filling the capillary pores and voids in the hardened cement paste located close to the surface of the reactive aggregate particles. This is also facilitated by the existence of the so-called interfacial transition zone (ITZ) that is a layer of material with higher porosity in the hardened cement paste near the aggregate surface (see Figure 1a). Similarly to the ITZ size, the equivalent thickness, $\delta_{c}$, of the layer in which the capillary pores are accessible to the ASR gel may be considered constant and independent of the particle size *D*. To account for this behavior, the amount of imbibed water used to compute the aggregate expansion is defined by $\langle M_{i}-M_{i}^{0}\rangle$, where $M_{i}^{0}=\left(4\pi \rho_{w}/3\right)\left({\left(r+\delta_{c}\right)}^{3}-r^{3}\right)$ is the mass required to fill this space, $\rho_{w}$ is the mass density of water, and the brackets 〈〉 extracts the positive value of the expression. The increased radius of each aggregate particle of initial radius r=D/2 can be calculated as $r_{i}={\left(3\langle M_{i}-M_{i}^{0}\rangle /4\pi \rho_{w}+r^{3}\right)}^{1/3}$. The rate of radius increase can be written using the chain rule as

(22) $${\dot{r}}_{i}=\frac{dr_{i}}{dt}=\frac{dr_{i}}{dM_{i}}\frac{dM_{i}}{dt}={\dot{M}}_{i}\frac{dr_{i}}{dM_{i}}=\frac{{\dot{M}}_{i}}{4\pi \rho_{w}}{\left(3\langle M_{i}-M_{i}^{0}\rangle /4\pi \rho_{w}+r^{3}\right)}^{-2/3}$$

This definition of radius change rate is used to compute an incompatible ASR gel normal strain rate as

(23) $${\dot{e}}_{N}^{a}=\left({\dot{r}}_{i1}+{\dot{r}}_{i2}\right)/\ell$$

where $r_{i1}$ and $r_{i2}$ are the increases in the radii of the two aggregate particles sharing a generic facet. Note that the model formulated herein assumes approximately that the imposed facet shear strains due to gel swelling are negligible, $e_{M}^{a}=e_{L}^{a}\approx 0$, although this might not be exactly true due to the irregular shape of actual aggregate particles. Based on this simplification, the ASR gel strain rate is given by:

(24) $${\dot{\epsilon }}^{a}={\left[\begin{array}{ccc} {\dot{e}}_{N}^{a} & 0 & 0 \end{array}\right]}^{T}$$

### 2.6. Thermal and Hygral Deformations

Most materials expand/shrink proportionally to temperature increase/decrease. The coefficient of proportionality is assumed to be a material property called coefficient of thermal expansion, $\alpha_{T}$. So, the thermal strain rate can be given by:

(25) $${\dot{\epsilon }}^{t}={\left[\begin{array}{ccc} \alpha_{T}\dot{T} & 0 & 0 \end{array}\right]}^{T}$$

Similarly, to account for hygral variation, one can write:

(26) $${\dot{\epsilon }}^{s}={\left[\begin{array}{ccc} \alpha_{h}\dot{h} & 0 & 0 \end{array}\right]}^{T}$$

and $\alpha_{h}$ is the so-called shrinkage coefficient which in typical situations is identified from drying tests. In the above formulations, $\alpha_{T}$ and $\alpha_{h}$ are assumed to be average concrete properties which represent average properties of aggregate and mortar.

## 3. Numerical Implementation

Numerical implementation of the concrete constitutive equations requires that at each step, the stress increment Δσ is calculated on the basis of the response at the previous step and current strain increment Δϵ. At the beginning of each step, prior to integrating the constitutive equations, the one way coupling between the chemo-physical model and the mechanical model is imposed directly at the facets centroids. The shape functions of the HTC tetrahedral mesh are first used to determine which facets lie inside each tetrahedron, next each facet is assigned an exchange function that uses the HTC tetrahedron shape functions to interpolate—at the facet centroid—the values of HTC nodal variables and their instantaneous rates (namely temperature *T*, temperature rate $\dot{T}$, relative humidity *h*, relative humidity rate $\dot{h}$, and cement hydration degree, $\alpha_{c}$). Figure 1e shows one of the cylinder and prism geometries used in simulations with modeled aggregate shown inside both and colored by their radii. As discussed before, around each aggregate, a set of facets is obtained. Figure 1f shows an 8th of the cylinder where both aggregate (in gray) and facets (in purple) are shown. Figure 1g shows the corresponding 8th of the HTC cylindrical mesh colored by the values from the humidity field and Figure 1h shows the same values interpolated on the facets.

For the integration of the constitutive equations to be explicit, all strain increments other than $\Delta \epsilon^{*}$ can be considered as imposed strain increments. From the rearrangement of Equation (18) in an incremental form one has:

(27) $$\Delta \epsilon^{*}=\Delta \epsilon -\left(\Delta \epsilon^{a}+\Delta \epsilon^{s}+\Delta \epsilon^{t}+\Delta \epsilon^{v}+\Delta \epsilon^{f}\right)$$

At the beginning of each time step, nodal velocities are used to evaluate the rates of displacement jumps at each LDPM facet, from which, the total facet strain rate $\dot{\epsilon }$ is computed. By simply multiplying it by Δt, the total strain increment becomes $\Delta \epsilon =\Delta t\dot{\epsilon }$.

Shrinkage $\Delta \epsilon^{s}$ and thermal $\Delta \epsilon^{t}$ strain increments are computed at each facet based on humidity and temperature increments at the beginning of the time step as $\Delta \epsilon^{s}=\alpha_{h}\Delta h{\left[1\;0\;0\right]}^{T}$ and $\Delta \epsilon^{t}=\alpha_{T}\Delta T{\left[1\;0\;0\right]}^{T}$.

For the ASR strain increment, at the beginning of each time step, all aggregate in which ASR is progressing are computed by first advancing the diffusion fronts through integrating Equation (9) over the time increment Δt using forward Euler method. Then, the gel masses are computed from Equation (1) and finally substituted in Equation (11). Again, the rate of imbibed water mass is integrated over Δt to give the current increment in imbibed water $\Delta M_{i}$. For each aggregate piece, the increase in radius is computed from the incremental form of Equation (22) as $\Delta r_{i}=\Delta M_{i}/\left(4\pi \rho_{w}\right){\left(3\langle M_{i}-M_{i}^{0}\rangle /4\pi \rho_{w}+r^{3}\right)}^{-2/3}$ . Then, the gel normal strain increment is computed as $\Delta e_{N}^{a}=\left(\Delta r_{i1}+\Delta r_{i2}\right)/\ell$ and the ASR imposed strain increment vector becomes $\Delta \epsilon^{a}={\left[\Delta e_{N}^{a}\;0\;0\right]}^{T}$.

Finally, also the creep strain increment is calculated on the facet level under the assumption of constant stress. This assumption means that the creep strain is integrated with a step-wise stress history in which the value of the current stress has a one time step delay, as done in the Euler explicit method for numerical integration of differential equations. In this case the global error is proportional to the step size, which, however, is very small due to the explicit numerical implementation of LDPM.

The viscoelastic creep strain is modeled as an aging multi Kelvin chain model. For a one dimensional single Kelvin model with spring constant $E_{j}$ and damper coefficient $\eta_{j}$ the stress σ is given by $\sigma =E_{j}\gamma_{j}+\eta_{j}{\dot{\gamma }}_{j}$, where $\gamma_{j}$ is the strain. Let $\tau_{j}=E_{j}/\eta_{j}$ be the retardation time constant of the Kelvin unit. Because the stress is assumed constant, $\sigma \left(t\right)=\sigma \left(t_{i}\right)=\sigma^{i}$, in the time step from $t_{i}$ to $t_{i+1}$ with $\Delta t=t_{i+1}-t_{i}$, the general solution of the strain evolution is given by $\gamma_{j}\left(t\right)=A+B\exp\left[-\left(t-t_{i}\right)/\tau_{j}\right]$ with $A=\sigma^{i}/E_{j}$ and $B=\gamma_{j}^{i}-\sigma^{i}/E_{j}$ (obtained imposing the initial condition $\gamma_{j}\left(t_{i}\right)=\gamma_{j}^{i}$). The strain at time $t_{i+1}$ is then given by

(28) $$\gamma_{j}^{i+1}=\frac{\sigma^{i}}{E_{j}}\left(1-e^{-\Delta t/\tau_{j}}\right)+\gamma_{j}^{i}e^{-\Delta t/\tau_{j}}$$

and the strain increment becomes

(29) $$\Delta \gamma_{j}^{i}=\left(\frac{\sigma^{i}}{E_{j}}-\gamma_{j}^{i}\right)\left(1-e^{-\Delta t/\tau_{j}}\right)$$

For a chain of *N* Kelvin elements we have

(30) $$\Delta \gamma^{i}=\sum_{j=0}^{N}\left(\frac{\sigma^{i}}{E_{j}}-\gamma_{j}^{i}\right)\left(1-e^{-\Delta t/\tau_{j}}\right)$$

Following [102], the non-aging compliance $1/E_{j}=A_{j}$ is computed for each chain to satisfy,

(31) $$A_{0}+\sum_{j=1}^{N}A_{j}\left(1-e^{-\Delta t/\tau_{j}}\right)\approx \xi_{1}\ln\left[1+{\left(\Delta t\right)}^{0.1}\right]$$

According to [102], logarithmically equally spaced values for $\tau_{j}$ are used to cover a wide range of creep response, ten elements are used with a retardation time ranging from $10^{-4}$ to $10^{5}$ days. This gives $A_{0}=0.279\xi_{1}\ln\left(10\right)$ for $\tau_{0}=0$ [99]. $A_{0}$ is the compliance of an elastic element that accounts for very short time creep (<10 min load duration) typical of quasi-static lab tests. With these values for $\tau_{j}$, $A_{j}=L_{j}\ln\left(10\right)$ and using an approximate retardation spectrum of order 3 [102], $L_{j}$ is given by

(32) $$\begin{array}{ccc} L_{j} & = & \frac{{\left(3\tau_{j}\right)}^{3}}{2}\xi_{1}\left[\frac{-2n^{2}{\left(3\tau_{j}\right)}^{2n-3}\left(n-1-{\left(3\tau_{j}\right)}^{n}\right)}{{\left[1+{\left(3\tau_{j}\right)}^{n}\right]}^{3}}\right.\\ \; & + & \left.\frac{n\left(n-2\right){\left(3\tau_{j}\right)}^{n-3}\left(n-1-{\left(3\tau_{j}\right)}^{n}\right)-n^{2}{\left(3\tau_{j}\right)}^{2n-3}}{{\left[1+{\left(3\tau_{j}\right)}^{n}\right]}^{2}}\right] \end{array}$$

Also by considering a constant $\psi \left(t^{i}\right)=\psi \left(t^{i+1}\right)=\psi^{i}$ over the time step, one can write, $\Delta t_{r}=t_{r}\left(t^{i+1}\right)-t_{r}\left(t^{i}\right)=\int_{0}^{t^{i+1}}\psi \left(\tau \right)d\tau -\int_{0}^{t^{i}}\psi \left(\tau \right)d\tau =\int_{t^{i}}^{t^{i+1}}\psi \left(\tau \right)d\tau =\psi^{i}\Delta t$. So, including all effects, the viscoelastic strain increment is given by

(33) $$\Delta \epsilon^{v}=\sum_{j=1}^{N}\left(GA_{j}\sigma^{i}-\gamma_{j}^{i}\right)\left(1-e^{-\psi^{i}\Delta t/\tau_{j}}\right)\frac{1}{v(\alpha_{c}^{i})}$$

Similarly, the purely viscous strain increment at the facet level is computed considering again constant stress $\sigma^{i}$, constant $\psi^{i}$ and similarly constant $\psi_{s}^{i}=\psi_{s}\left(t^{i}\right)$, in the time step Δt. This gives:

(34) $$\Delta \varepsilon^{f}=\Delta t\xi_{2}\kappa_{0}\psi^{i}S^{i}G\sigma^{i}$$

with the following relation to update the microprestress *S*,

(35) $$\Delta S^{i}=-\psi_{s}^{i}\kappa_{0}S^{i^{2}}\Delta t+\kappa_{1}\left|\Delta T^{i}\ln\left(h^{i}\right)+T^{i}\frac{\Delta h^{i}}{h^{i}}\right|$$

By subtracting the imposed strain increments, the remaining will be the strain increment $\Delta \epsilon^{*}$ which is used by the LDPM constitutive law to compute the corresponding facet stress vector increment Δσ and update the stress vector at the end of the time step. The LDPM equations are integrated with reference to the apparent normal modulus ${\bar{E}}_{0}\left(t\right)$ defined as:

(36) $${\bar{E}}_{0}\left(t\right)=\frac{1}{\frac{1}{E_{0}}+\frac{A_{0}}{v(\alpha_{c}\left(t\right))}}$$

This means that the incrementally elastic effective LDPM stress (see Section 2.3.1) is calculated at each step as $\Delta t^{el}={\bar{E}}_{0}\left(t_{i}\right)\Delta e$. The nonlinear part of the LDPM constitutive equations is imposed through a vertical return algorithm [67].

The presented formulation, is implemented into MARS, a multi-purpose computational code for the explicit dynamic simulation of structural performance [103].

## 4. Numerical Simulations and Comparison with Experimental Data

This section presents numerical simulations of experimental data relevant to concrete specimens and structural members with and without reinforcement undergoing ASR deformations in different environmental conditions as presented in Reference [104]. Three sets of experiments were performed. The first and second sets were performed using cylindrical specimens (320 mm length and 160 mm in diameter). The first set included uniaxial compression tests and Brazilian splitting tests to characterize concrete strength and stiffness. In the second set, the tests performed were free ASR expansions lasting 480 days from casting. Three different relative humidity conditions were considered: (1) 100% RH (saturation); (2) completely sealed; and (3) 30% RH, and both mass changes and total axial deformations of specimens were reported. The third set was relevant to the structural member scale. In this set, full scale (3 m long and 250×500 mm cross-section) simply supported beams were instrumented to collect their long term deformation over 420 days after being cured in sealed conditions for 28 days. Beam internal humidity profile was measured at 4 locations along the beam depth. No external load other than the self weight of the beam was applied. Beams were kept at a slightly elevated temperature of 38 ${}^{\circ }C$ with both lateral sides sealed with aluminum sheets. A nearly 1D humidity profile was created along the beam by immersing its bottom 7 cm in water and leaving the top surface exposed to a controlled relative humidity of 30%. Five different beams were tested: 2 were nonreactive control beams with and without reinforcement (labeled here as NPC and NRC, respectively); one reactive plain concrete (labeled here as RPC) beam and two reactive reinforced concrete beams with different (0.45% and 1.8%) longitudinal reinforcements (labeled here as RRC1 and RRC2, respectively).

**Figure 2:**
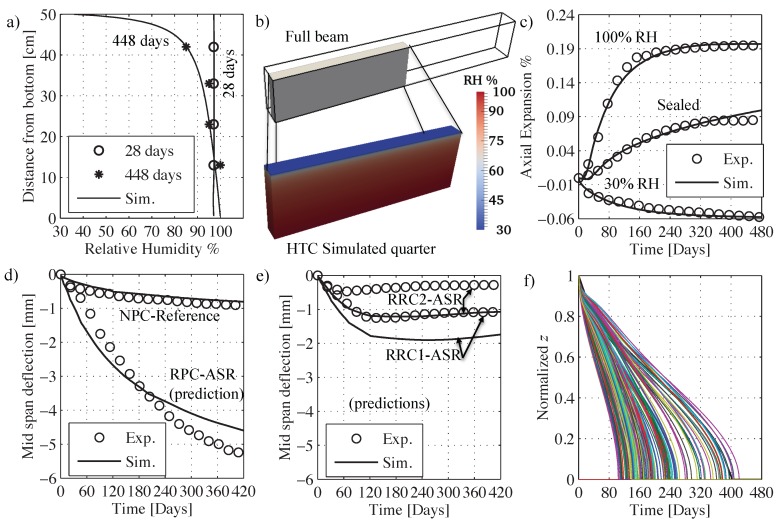
(**a**) Experimental and numerically simulated RH values along the depth of the beam at 28 and 448 days; (**b**) HTC mesh colored by the RH field at 448 days showing the quarter that was simulated; (**c**) Experimental and numerically simulated average axial expansions of both cylinders and prisms under fully saturated, sealed and 30% RH exposure conditions; (**d**) Midspan deflections of unreinforced NPC and RPC beams; (**e**) Midspan deflections of reinforced RRC1 and RRC2 beams; (**f**) Normalized evolutions of all simulated aggregate diffusion fronts.

**Figure 3:**
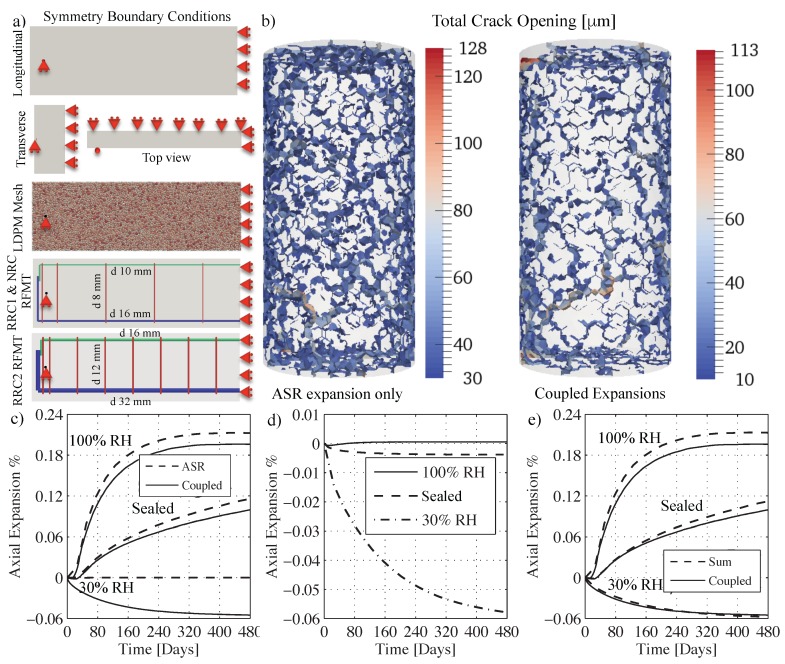
(**a**) Beam simulated geometry, showing symmetry boundary conditions, LDPM generated mesh and reinforcements for NRC, RRC1 and RRC2 beams (Aggregate are colored by their relative size); (**b**) Simulated crack pattern distribution due to ASR with coupling and without coupling with creep and shrinkage deformations; (**c**) Simulated pure ASR expansion versus coupled ASR, creep and shrinkage expansion; (**d**) Simulated creep and shrinkage expansions only; (**e**) Sum of simulated ASR shrinkage and creep expansions versus fully coupled expansion.

The generated geometries used in the numerical simulations consisted of two types: FE meshes for the HTC model and particle systems for LDPM. Both specimens (cylinders and prisms) and beams were discretized. All HTC model meshes made full use of any possible axes of symmetry (X,Y and Z for both prisms and cylinders and X a Y only for beams) which resulted in meshing only 1/8 of both cylinders and prisms and meshing 1/4 of the beams. As for the LDPM systems, all cylinders and prisms were fully meshed, but as the beams were taking a huge computational time, symmetry was used also for the LDPM beam specimens. As will be explained in the discussion of results, 1/8 LDPM samples were also generated and ran with the same full samples parameters to check if there was any significant effect of applying symmetry boundary conditions on the heterogeneous LDPM system. Figure 1e shows the LDPM cylinder and prism meshes and Figure 1g shows the HTC mesh for the cylinder. HTC and LDPM beams are also shown in Figure 2b and Figure 3a, respectively.

### 4.1. Identification of Cement Hydration Parameters

The experimental data did not include relevant tests to identify these parameters. Hence, they were assumed based on existing literature and they are reported in Table A1.

### 4.2. Identification of HTC Parameters

The relative humidity measurements from the NPC beam were used to calibrate the HTC model parameters. The 4 sensors placed at 8, 17, 27 and 37 cm from the top drying surface of the beam recorded RH = 97% after 28 days of sealed curing; whereas after 14 months, the top one recorded RH = 85%, the lower one RH = 100% and the two middle ones RH ≈ 95%. These values were used for the HTC model calibration. The identified parameters are listed in Table A3 along with values of other parameters that were assumed on the basis of existing literature.

Figure 2a shows excellent agreement between the simulated humidity profile and the reported sensor data. It must be considered here that most of the relative humidity sensors have an error of about 1% to 2% in the middle range of relative humidity (20% to 80%) and around 2% to 4% close to saturation and dry conditions. Figure 2b shows the HTC mesh for one quarter of the beam, colored by relative humidity at 14 months from curing (448 days).

### 4.3. Identification of Shrinkage and Creep Parameters

Given the HTC parameters, the internal relative humidity change in the cylinder kept in an environment with 30% of relative humidity is known. So, its axial deformation history can be used to identify the shrinkage coefficient $\alpha_{h}$. This gives $\alpha_{h}=$ 9$\times 10^{-4}$ which is in excellent agreement with typical values reported in the literature [96]. Simulated vs experimental deformation curves are shown in Figure 2c and the two curves are nearly identical. The simulation results are the average of both cylinders and prisms axial deformations at 30% relative humidity exposure, while the experimental curve is the cylinder axial deformation only as no prism deformations were reported at 30% in Reference [104]. Table A4 reports the hygro-thermal parameters used.

Identifying creep parameters from only one drying creep curve is a challenging task. The reported deformations are measured after 28 days of curing and no clear information are provided about the supporting condition during curing, so it was assumed that the beams were resting on ground. In addition, it is not clear when the first deflection was measured after loading. These factors create uncertainty in the creep data in the early stage of loading. Therefore, the reported quasi-static elastic modulus was used to calibrate the parameter $E_{0}$. The meso-scale creep compliance at 28 days of age and 0.001 load duration can be assumed to be equal to the reciprocal of the apparent LDPM normal modulus at 28 days ${\bar{E}}_{0}^{28}={\bar{E}}_{0}$(28 days)=1/J(28,0.001) as typically accepted in the literature [105,106]. In addition, it can be assumed that $\xi_{1}\approx 2.3/E_{0}$ based on average ratios of their values in the extensive calibrations presented in Reference [96]. With this assumption, three independent parameters $E_{0}$, $\xi_{2}$ and $\kappa_{1}$ need to be calibrated using 2 different tests. The first test is the simulation of the apparent elastic modulus according to the ASTM C469 method [107]. In this test, the secant modulus at 45% peak load is used for calibration. For the elastic modulus test, the contribution of the viscous creep part during the 0.001 day loading time is very small, thus it is mainly calibrating $E_{0}$. The second test is the simulation of the NPC beam mid-span deflection history. For this test, the slope of the long term creep deformation is mainly governed by $\xi_{2}$, therefore, although only 2 tests are used to calibrate 3 independent parameters, the test data allow a unique identification calibrate because not all the three parameters affect each part of the tests equally.

Following this procedure, the calibrations yielded $E_{0}=$ 133.33 GPa, $\xi_{1}=$ 1.75 $\times 10^{-5}$ MPa${}^{-1}$, $\kappa_{1}=$ 19 MPa${/}^{\circ }K$ and $\xi_{2}=$ 7 $\times 10^{-6}$ MPa${}^{-1}$. All creep parameters are listed in Table A2. The simulated elastic modulus was $E_{num}^{28}=37.7$ GPa and the experimentally reported value was $E_{exp}^{28}=37.3$ GPa which means that the error is less than 1.07%. Figure 2d shows the relevant experiments versus simulations comparison. The numerical results match well the deformation trend and magnitude over most of the time history up to the end. Only the early part is slightly underestimated at about 2 months. Many factors affect this difference including the estimation of early age creep parameters for lack of specific experiments and possible shortcomings in the experiments. The latter includes imperfect sealing, accuracy of humidity profile measurement, and the general variability of lab results for concrete testing. It is also worth mentioning that the experiments on beam specimens had only one beam sample per case which, of course, has limited statistical validity.

### 4.4. Calibration of LDPM Concrete Parameters

LDPM parameters were calibrated based on reported values of compressive strength, $f_{c}^{\prime }=$ 38.4 MPa, and splitting tensile strength, $f_{t}^{\prime }=$ 3.2 MPa. The generation of the different LDPM meso-structures was performed considering the aggregate size distribution reported in Reference [108]. The parameters used for geometry and aggregate system generation were: minimum aggregate size, $d_{0}=10$ mm; maximum aggregate size, $d_{a}=20$ mm; fuller curve exponent, $n_{F}=0.79$; cement content, c=410 $\mathrm{kg}/m^{3}$; water-to-cement ratio, w/c=0.5207; aggregate-to-cement ratio, a/c=4.249.

The identified LDPM parameters were: meso-scale tensile strength, $\sigma_{t}=4.75$ MPa; shear strength ratio, $\sigma_{s}/\sigma_{t}=3.07$; and meso-scale tensile characteristic length, $\ell_{t}=75$ mm. Other parameters were assumed based on existing literature and they are listed in Table A2. The average of the simulated concrete properties are: $f_{c,num}^{\prime }=38.41$ MPa and $f_{t,num}^{\prime }=3.19$ MPa, which match the given experimental data with an error smaller than 0.026% and 0.31%, respectively.

### 4.5. Calibration of ASR Model Parameters

The already calibrated HTC, creep, shrinkage and LPDM parameters are used with no changes and coupled with ASR response during the ASR parameter calibration step. This is the only reasonable approach for a calibration process that represents realistic ASR evolution because the visco-elastic character of the model can render the simulated ASR expansion unreliable by under predicting it (if the compliance is too high) or over predicting it (if the compliance is underestimated). Similarly, the HTC model parameters are extremely important because they characterize the *h* and *T* fields that affect ASR processes. It is worth mentioning that the identified ASR parameters are relevant to *T* = 38 ${}^{\circ }C$, which was the temperature at which the tests were performed.

The identification is here executed in two main stages: stage I concerns the calibration of ASR evolution parameters at full saturation; stage II deals with the identification of the parameters governing the effects of relative humidity on ASR induced expansion.

*Stage I: Calibration under 100% relative humidity.* The calibration first step is to try decoupling the two ASR processes, namely gel formation and water imbibition. The evolution rate of both processes decreases with time: the gel formation slows down and stops as the aggregate becomes fully reacted and ${\dot{M}}_{i}$ is proportional to the difference between the mass of imbibed water, $M_{i}$, and the thermodynamic imbibition capacity. Thus it is possible to fit an ASR expansion curve by over- or under- estimating one process rate and doing the opposite with the other one especially if the ASR expansion curve does not reach an asymptotic value within the experimental testing period. By examining the axial deformation curves for the 100% case for both cylindrical and prismatic samples in Reference [104], it is clear that they reach a plateau at about 420 days. So, while calibrating ASR parameters, a check was made to have the largest aggregate pieces react completely around 420 days. Since the temperature is constant throughout the test period, only $\kappa_{z}^{1}$ needs to be calibrated to adjust the time of full aggregate reaction. Hence, regardless of matching the expansion curve amplitude, $\kappa_{z}^{1}=$ 2.62 $\times 10^{-10}$ m${}^{5}/$(kg day) was directly obtained. In the actual experiments, the fine aggregate was not reactive while the coarse aggregate (>4 mm in diameter) was reactive therefore all reactive aggregate could have been modeled in LDPM. The problem is that, while doable for small samples, it is too expensive for large sample size. Therefore, a cut off radius is usually used in all LDPM simulations. The usual limit [66,67] is to assume $d_{min}=0.5d_{max}$ which was used in the calibration of LDPM parameters as mentioned in Section 4.4. The only problem here is that the expansion from smaller aggregate that would be cut off needs to be accounted for. It is important here to say that the coarser aggregate has more significance in cracking than the fine aggregate as it produces more gel over longer times. Figure 2f shows the normalized diffusion front profiles of all simulated aggregate. By examining Figure 2f, the smallest modeled aggregate (d= 9.89 mm) completely reacted after only 120 days, so, the coarser aggregate alone is responsible of the heterogeneity in expansion (which is the main reason for cracking [65]) from 120 to 480 days.

Next, the amplitude of the expansive deformation due to ASR and its profile also need to be fitted. In the model, its initial part is controlled by $\delta_{c}$ and ${\tilde{C}}_{i}^{1}$ while the amplitude is controlled by $\kappa_{a}\times \kappa_{g}\times \kappa_{i}$. In the reference experimental program [104], potassium hydroxide was added to the mixing water to raise the alkali content to 1.25% by cement mass of Na${}_{2}$O${}_{\mathrm{eq}}$ as typically done in similar accelerated tests for ASR [109,110]. Thus $c_{a}=c\times 1.25/100=410\times 1.25/100=5.125$ kg/m${}^{3}$. This value is typically higher than the required saturation alkali content [65]. Therefore, the available alkali content is more than enough to react with all silica in aggregate. This leads to $\kappa_{a}=1.0$. Furthermore, as the gel composition and silica content are not known from Reference [104], a reasonable estimate of $\kappa_{g}=$689 kg/m${}^{3}$ can be obtained based on previous works [65,68]. This means that only $\kappa_{i}$, $\delta_{c}$ and ${\tilde{C}}_{i}^{1}$ are free parameters. The calibration now is simple, first, $\delta_{c}$ is set to zero, then, an initial estimate of $\kappa_{i}$ is obtained. Next, ${\tilde{C}}_{i}^{1}$ is calibrated to match the linear slope of the middle stage of ASR expansion. Finally, to match the initial delay along with the final asymptote, $\delta_{c}$ is introduced along with adjusting $\kappa_{i}$. Then fine tuning is done for the three parameters. It must be noticed here that none of the parameters can interfere with the identification of the other two, and basically, each parameter adjusts a specific portion of the full ASR expansion curve. This final step gives $\delta_{c}=$6.0$\times 10^{-6}$ m, ${\tilde{C}}_{i}^{1}=$7.78 $\times 10^{-10}$ m${}^{2}/$day, and $\kappa_{i}=$ 1.45 $\times 10^{-2}$.

It must be mentioned here that the experimental data are largely scattered, therefore, the calibrations, discussed above, were performed on the average axial deformation of cylinders and prism samples. For more details on the reasons of this scatter, one can refer directly to Reference [104], in which the main explanation was the different direction of concrete casting for prisms and cylinders.

*Stage II: Calibrating relative humidity effects parameters.* At this point, only two parameters remain to be calibrated $r_{D}$ and $n_{D}$. For this calibration, the average of experimental data for sealed samples (both cylinders and prisms) is used. The calibrated parameters are $r_{D}=$ 3600 and $n_{D}=$ 2. It must be noted here that the sealed samples had a relative humidity of 97% at 28 days. At this value, and using the calibrated parameters, the diffusivity ratio becomes $1/(1+\left(3600-1\right){\left(1-0.97\right)}^{2})=0.236$. This means that calibrating at 97% relative humidity is covering a wide range of the humidity effect. The fitted expansive deformation due to ASR is shown in Figure 2c. It is worth noting also that the experimental program suffered from small water loss in the sealed samples as reported by Reference [104], therefore, the slightly increasing final value in the numerical simulations that does not match the average can be explained by that moisture loss in the experiments. Nevertheless, this slight difference is way below the experimental scatter for sealed specimens, where the final expansion range was from 0.25% to 1.36%.

At this point, all models parameters are fully calibrated, all effort was made to minimize redundancy and to keep the calibration process as uncoupled as possible. All ASR parameters are listed in Table A5. Now, it only remains to validate the overall framework against a completely different scale and range of conditions which is left for the next section.

### 4.6. Validation through Full Scale Beam Simulations

The predictive capabilities of the framework can be verified through the simulation of a set of experiments not used in the calibration phase and relevant to the same concrete material utilized. The set consists of 3 different reactive beams tested in Reference [104] with the same dimensions of the NPC beam used in the creep model calibration. Figure 3a shows the geometry of the beams along with their reinforcement. As can be seen from Figure 2d, a good matching between experimental and simulated responses is achieved for the RPC beam. In the beginning, simulations tend to over estimate the response but then, towards the end, the response is underestimated where the experiments showed 5.2 mm deflection while the simulated one was 4.6 mm which is just 12% smaller. In fact, this is an excellent prediction given that the scatter observed in experimental data used for ASR calibration was over 20%. As for the reinforced beams RRC1 and RRC2, the model correctly captures the different stages of the response: and it shows an increase in deflection in the beginning; then as time goes on, it starts to plateau, then finally, the deflection decreases back. This is because the lower saturated region reacts faster and more than the middle region. In addition, the top region tends to shrink due to drying. The combined effect is generation of a curvature that leads to initial downward deflection for the samples RPC, RRC1, and RRC2. For RPC, since no reinforcement is present, the ASR induced expansions in the bottom part are at their maximum and shrinkage of the top is also unrestrained, as a result, the beam bends down and never returns up again, but towards the end, the deflection rate slows down as both shrinkage and ASR induced deformations reach a plateau. For RRC1 and RRC2, the presence of reinforcement constrains top and bottom deformations. Especially in the bottom where the reinforcement area is 2.25 times the top one for RRC1 and 4 times the top one for RRC2, the overall ASR induced expansion and thus the deflection is clearly reduced. However, in those samples the less restrained middle part starts to be of more relevance here as it keeps expanding towards the end of the test period. Therefore, the deflection of the beam does not show a plateau, and, instead, it cambers back up. This is much more clear with the deflection of the sample RRC2, which tends to camber up earlier than RRC1 (see Figure 2d). This is because, for RRC2, the bottom part is even more restricted compared to the top part than for beam RRC1. The model captures all these aspects, which means that it does represents the correct effects of humidity on ASR expansion, even if it over estimates the whole curve. The discrepancy can be partially explained again by the very large scatter in the experimental data and by the fact that only one beam sample of each type was tested. In addition, any slight change in the actual location of the reinforcement or possible slippage due to ASR cracking in the bottom part can also have an effect on the deflection.

## 5. Discussion of Results

As shown in the previous section, the proposed framework was able to replicate full structural members deformations induced by ASR under varying environmental conditions, loads, and reinforcement arrangements based on small companion specimens behavior. This is an unprecedented predictive capability that—to the best knowledge of the authors—has never been achieved before by any existing comprehensive and physics-based ASR model as they had to be calibrated on the actual structural member behavior to be able to replicate it, or they were only replicating special uniform conditions on lab specimens. Simplified empirical models that can only estimate deformations without explicit evaluation of damage and stress transfer, can only be used for structural type preliminary calculations. They can not be used to evaluate strength degradation and service life prediction. In fact, the power of this proposed framework is not limited to its predictive capability, but it extends to the ability to see inside the structural element and to understand more in details how different phenomena interact. In this section, emphasis on understanding these coupling aspects is presented to elucidate the unseen redistribution of cracking and stress relief as a result of creep-ASR coupling.

First, by looking closely at Figure 3b, with a 30 μm crack opening cutoff, it is pretty clear that in the case of considering only ASR effect the specimen presents much more distributed small cracks and the maximum crack opening is 128 μm. Whereas in the case of fully coupled model (ASR expansion with creep and shrinkage) the specimen presents less distributed cracks (about 13% less cracking) with a maximum crack opening of 113 μm. Figure 3c shows, for the fully coupled model and the ASR-only, the axial deformations versus time obtained under different conditions, namely 100% environmental RH, sealed condition, and 30% environmental RH. For the 100% RH case, the axial expansion due to ASR-only is 0.2129% while it is 0.1962% for the coupled case with a difference of 8.5%. For the sealed case, the axial expansion with ASR-only is 0.1158% compared with 0.0995% for the coupled model. In this case the effect of coupling is more pronounced with a significant difference of 16.4%. This is partially due to the slight shrinkage caused by self-desiccation in sealed condition that is opposite to the slight swelling caused by resaturation for the 100% RH case. Finally, as a proof that ASR does not significantly affect the calibration of the shrinkage coefficient based on the 30% RH case axial deformation, the simulated expansion with ASR-only model at 30% RH was only 1.27$\times 10^{-4}$%.

To further understand the actual contributions to the observed deformation, the axial deformations were also simulated for the three different cases and are plotted in Figure 3d. At 100% RH swelling is very small (only 5.6$\times 10^{-4}$%) but a little shrinkage is observed in the sealed case which was −3.8$\times 10^{-3}$%. If this is subtracted from the coupled case, the sealed expansion becomes 0.1033% and still the uncoupled ASR expansion is 12% higher than the coupled one. This means that for the sealed case, although the overall expansion is less, the creep affects more the overall deformation. This can be explained again in a fully coupled setting because, as the relative humidity drops, the microprestress decay is slowed down slightly and thus, more viscous strains can develop. In addition, the higher ASR imposed strains in the 100% RH case cause earlier cracking which, in turn, prevents these cracks from contributing to creep/relaxation of the internal stresses build up. It is very important here to notice that, if a continuum based formulation is used, all these meso-scale phenomena can not be explicitly captured and, on the contrary, they have to be phenomenologically assumed. Thanks to the discrete setting and the mimicking of concrete internal structure and heterogeneity, this framework allows for clear understanding of the coupling mechanisms and their interactions. In fact, almost all other available models add shrinkage/swelling expansions algebraically to ASR expansions without any consideration of creep as they are all continuum based and in case of free expansions, the macroscopic stress tensor is always zero. Only the model by Bažant and Rahimi-Aghdam [10] considers creep induced deformations as function of ASR induced pressure, but as mentioned before, it is done in an average sense, where the ASR induced pressure does not correspond directly to the actual aggregate-aggregate stress fields and thus, the estimated creep also does not directly correlate to the actual meso-scale level creep deformation. As a final clarification here, the sum of shrinkage/swelling and ASR deformations is compared to their corresponding result of the coupled model in Figure 3e and, again, at 100% RH the sum overestimates the coupled one by 8.8%, the sealed one is 12.6% overestimated, and at 30% RH no large difference is observed.

**Figure 4:**
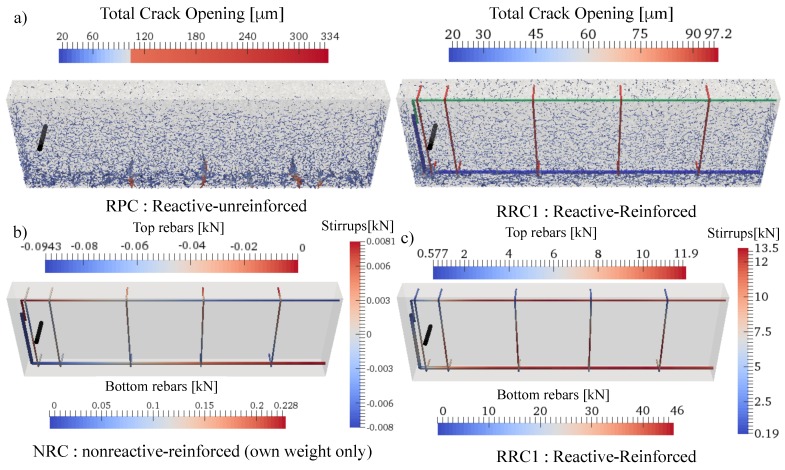
(**a**) Simulated crack patterns and crack openings for both RPC and RRC1 beams showing the effects of reinforcement on crack suppression; (**b**) Simulated rebar internal forces due to beam own weight only; (**c**) Simulated rebar internal forces due to beam own weight, Alkali Silica Reaction (ASR), creep and shrinkage effects.

This becomes much more interesting when beam simulations are studied. First, the crack distributions are shown for RPC and RRC1 in Figure 4a from which it can be seen the difference of the cracks distribution and how the reinforcement confinement tends to reduce the amount of cracking and crack opening (334 μm for beam RPC and only 97 μm for beam RRC1). In Figure 4a the color scale of crack openings was intentionally modified for the RPC beam to show all cracks above 100 μm in red so they can be distinguished from those in RRC1. By looking at Figure 4b, the tensile forces along the rebars for NRC beam are plotted just after the application of its own weight (no creep nor drying is included). The bottom rebars are in tension with 0.228 kN per each 16mm bar and the top are in compression with 0.094 kN per bar. The beam self-weight is 23.6×0.25×0.5=2.95 kN/m which generates a mid span bending moment of 2.89 kNm (over a span of 2.8 m). Even neglecting any steel contribution (RPC case), the bottom fiber tensile stress is 0.283 MPa which is clearly below the concrete tensile strength by about an order of magnitude. After including ASR effect as shown in Figure 4c, the compression in top bars completely reversed into tension, 11.9 kN, almost constant along the rebar up to the beam end and it only decreases close to the support where the presence of more stirrups provides confinement, reduces expansion and, thus, reduces rebar tension. With ASR effects, the bottom bar force increased up to 46 kN which corresponds to a stress of 230 MPa that would have yielded the bottom reinforcement if mild steel was adopted. In addition, this high level of stress means clearly that rebar-concrete slippage probably occurred in the experiments. While LDPM was recently extended to capture bond-slip behavior [111], due to lack of enough experimental data, this phenomenon was neglected in this study. The possibility of slippage supports the explanation of why the simulated RRC1 and RRC2 deflections are larger than the experimental ones. If slippage had occurred in the simulations, the stresses would have been relieved, the curvature would have been smaller, and, consequently, deflections would have been smaller since most of the deflection is due to the rebar restricted ASR expansion as opposed to the very small applied own weight.

Figure 4b,c show the forces in both vertical and horizontal parts of the stirrups. The stirrups in NRC beam (under own weight only) shown in Figure 4b have minimal forces as expected. Close to the midspan, the top and bottom segments of the stirrups are the most stressed ones with −0.008 kN at the bottom (compression) and 0.008 kN at the top (tension) which is a result of the lateral strain due to Poisson effects. Figure 4c shows the forces in RRC1 beam where expansion due to ASR produces tension in the horizontal segments at the bottom and shrinkage due to top surface drying reduces that expansion. The top segments now carry a 2 kN and the bottom ones carry about 8 kN. In addition, the vertical segments are all in tension and carry a maximum of 13.5 kN. Again, this is 268 MPa of tensile stresses, which is only elastic for the used high strength steel (mild steels like A36 yield at 220 MPa). To conclude, the proposed framework was able to compute internal forces in the reinforcement, that can not be measured and extremely hard to theoretically estimate by properly coupling different mechanisms at the main concrete heterogeneity length scale.

## 6. Conclusions

In this paper a multi-scale multi-physics framework that simulates coupled ASR damage, thermal, shrinkage and creep deformations in concrete is presented. The framework accounts for variations in environmental conditions including temperature and moisture changes as well as concrete aging as a function of cement hydration. All phenomena are translated into imposed strains, that are applied to the Lattice Discrete Particle Model which simulates concrete mechanical behavior including cracking and damage in a discrete setting at its meso-scale (length scale of large aggregate pieces). The framework was fully calibrated based on small samples experimental data. Full scale plain concrete and reinforced concrete beams were simulated as a validation step. The obtained results suggest the following conclusions.

1. ASR progression is a process that takes a few years to multi-decades depending on moisture and temperature conditions as well as cement chemistry and aggregate mineralogy. This makes ASR in full interaction with other aging and deterioration phenomena like creep, shrinkage and thermal expansions. Simple addition of the deformation induced by these phenomena is incorrect because the different phenomena are nonlinearly coupled.
2. Meso-scale modeling reveals the sub-scale interactions between coupled phenomena that are not seen at the macroscopic length scale. Namely, for the case of ASR induced free expansion, only modeling of deformations at the meso-scale can capture meso-scale creep deformations and relaxation of meso-scale stress build up that are not seen at the macroscopic scale because the macroscopic stress is zero.
3. Relative humidity effect on ASR expansion is essentially a moisture diffusion controlled process that can be modeled similarly to relative humidity effects on moisture diffusivity in concrete.
4. Simplified average section models that describe creep and shrinkage can lead to large inaccuracy in predicting ASR deformations for nonsaturated conditions. The humidity profile has a significant effect on ASR expansions that can not be averaged.
5. ASR expansions in reinforced concrete elements can lead to large internal forces build up and may lead to reinforcement yielding, reinforcement slippage, and partial bond loss.
6. For any complex framework to be predictive, its calibration needs to depend on uncoupled phenomena, then, it must be validated clearly. This was accomplished here by a multi-step calibration procedure on companion specimens with no ASR expansion, followed by ASR expansion calibration, then finally validation on full scale beams. A key factor here is the degree of scatter in the experimental data which is reflected directly in the prediction results of the model.
7. To the best knowledge of the authors, this is the only framework in literature that was calibrated on individual lab size specimens and was able to predict structural behavior. Other models are either directly calibrated based on structural response to simulate structural behavior, or are calibrated and validated based on individual lab size specimens.

## Appendix A. Model Parameters

**Table A1:** Parameters to Simulate Concrete Chemical Reactions.

Param. (Units)	Modeled Chemical Reaction	Test for Calibration	Value	Source
$A_{c1}$ (h${}^{-1}$)	Cement hydration	Calorimetric tests	1.41$\;\times \;10^{7}$	[79]
$A_{c2}$ (-)	Cement hydration	Calorimetric tests	5$\;\times \;10^{-3}$	[79]
$\eta_{c}$ (-)	Cement hydration	Calorimetric tests	8	[79]

**Table A2:** Parameters to Simulate Concrete Mechanical Behavior.

Param. (Units)	Modeled Behavior or Mechanism	Test for Calibration	Value	Source
$E_{0}$ (GPa)	Elasticity	Any in the linear range	133	Calibrated
α (-)	Poisson’s effect		0.25	[67]
$\xi_{1}$ (MPa${}^{-1}$)	Short term visco-elasticity	Basic creep tests with short load duration (≈1 year)	1.75 $\times 10^{-5}$	Calibrated
$\xi_{2}$ (MPa${}^{-1}$)	Long term viscocity	Basic creep tests with long load duration (≈10 year)	7 $\times 10^{-6}$	Calibrated
$n_{\alpha }$ (-)	Aging visco-elasticity	Basic creep tests at different age of loading	1.9	[96]
$\kappa_{1}$ (MPa${/}^{\circ }K$)	Evolution of the microprestress at variable temperature and relative humidity	Drying creep test or transitional thermal creep tests	19	[96]
$\sigma_{t}$ (MPa)	Tensile fracture	Fracture tests or tensile strength tests	4.75	Calibrated
$\ell_{t}$ (mm)	Tensile fracture	Fracture tests or size effect tests	75	Calibrated
$\sigma_{s}/\sigma_{t}$ (-)	Cohesive behavior in shear	Unconfined compression test	3.07	Calibrated
$\mu_{0}$ (-)	Frictional behavior	Triaxial compression tests at low confinement	0.2	[67]
$\sigma_{N0}$ (MPa)	Frictional behavior	Triaxial tests at high confinement	600	[67]
$\sigma_{c0}$ (MPa)	Yielding and pore collapse	Hydrostatic compression test	150	[67]
$H_{c0}/E_{0}^{28}$ (-)	Yielding and pore collapse	Hydrostatic compression test	0.3	[90]
$H_{c1}/E_{0}^{28}$ (-)	Yielding and pore distorsion	Passively confined tests	0.1	[90]
$\kappa_{c0}$ (-)	Material densification after pore collapse	Hydrostatic compression test	4	[90]
$E_{d}/E_{0}^{28}$ (-)	Material densification after pore collapse	Hydrostatic compression test at very high pressure or with unloading	1	[67]

**Table A3:** Parameters to Simulate Heat Transfer and Moisture Transport in Concrete.

Param. (Units)	Modeled Behavior or Mechanism	Test for Calibration	Value	Source
$c_{t}$ (J/kg${}^{\circ }$C)	Heat transfer	Thermal conductivity tests	1100	[79]
$\lambda_{t}$ (W/m${}^{\circ }$C)	Heat transfer	Thermal conductivity tests	2.3	[79]
$g_{1}$ (-)	Moisture content	Sorption/desorption isotherms relevant to two different values of hydration degree	1.7	[79]
$g_{2}$ (-)	Moisture content in C-S-H pores	Sorption/desorption isotherms relevant to two different values of hydration degree	0.2	[79]
$D_{0}$ (kg/mm h)	Moisture transport	Humidity profile during drying tests	2 $\times 10^{-9}$	Calibrated
$D_{1}$ (kg/mm h)	Moisture transport	Humidity profile during drying tests	4 $\times 10^{-7}$	Calibrated
*n* (-)	Moisture transport	Humidity profile during drying tests	2.35	Calibrated

**Table A4:** Parameters to Simulate hygro-thermal deformation.

Param. (Units)	Modeled Behavior or Mechanism	Test for Calibration	Value	Source
$\alpha_{h}$ (-)	Shrinkage and swelling due to moisture content change	Shrinkage tests	9$\times 10^{-4}$	Calibrated
$\alpha_{T}$ (-)	Thermal expansion and contraction	Thermal expansion tests	1$\times 10^{-5}$	[96]

**Table A5:** Parameters to Simulate Alkali Silica Reaction in Concrete

Param. (Units)	Modeled Behavior or Mechanism	Test for Calibration	Value	Source
$\rho_{g}$ (kg/m${}^{3}$)	ASR gel density	Free ASR expansion tests at 100 % relative humidity	689	[65,68]
$\kappa_{z1}$ (cm${}^{5}$kg${}^{-1}$day${}^{-1}$)	ASR gel formation	Free ASR expansion tests at 100 % relative humidity	2.62	Calibrated
$\kappa_{i}$ (-)	Water imbibition	Free ASR expansion tests at 100 % relative humidity	1.45 $\times 10^{-2}$	Calibrated
${\tilde{C}}_{i}^{1}$ (mm${}^{2}$/day)	Water imbibition	Free ASR expansion tests at 100 % relative humidity	7.78	Calibrated
$\delta_{c}$ (μm)	ITZ porosity effect on ASR imposed strain	Free ASR expansion tests at 100 % relative humidity	6.0	Calibrated
$c_{a0}$ (kg/m${}^{3}$)	Alkali content effect	Free ASR expansion tests at 100 % relative humidity and different alkali contents	2.7	[65]
$c_{a1}$ (kg/m${}^{3}$)	Alkali content effect	Free ASR expansion tests at 100 % relative humidity and different alkali contents	4.37	[65]
$r_{D}$ (-)	Relative humidity effect	Free ASR expansion at different levels of relative humidity	3600	Calibrated
$n_{D}$ (-)	Relative humidity effect	Free ASR expansion at different levels of relative humidity	2	Calibrated
